# Does whole-body bone SPECT/CT provide additional diagnostic information over [18F]-FCH PET/CT for the detection of bone metastases in the setting of prostate cancer biochemical recurrence?

**DOI:** 10.1186/s40644-020-00333-y

**Published:** 2020-08-12

**Authors:** Nicolas de Leiris, Julien Leenhardt, Bastien Boussat, Christopher Montemagno, Alexandre Seiller, Olivier Phan Sy, Julie Roux, Mathieu Laramas, Camille Verry, Carole Iriart, Gaelle Fiard, Jean-Alexandre Long, Jean-Luc Descotes, Jean-Philippe Vuillez, Laurent Riou, Loïc Djaileb

**Affiliations:** 1 Nuclear Medicine Department, Grenoble Alpes University Hospital, Grenoble, France; 2 INSERM, U1039, Radiopharmaceutiques Biocliniques, Grenoble, France; 3 Public Health Department, Grenoble-Alpes University Hospital, Grenoble, France; 4 Department of Oncology, Grenoble Alpes University Hospital, Grenoble, France; 5 Department of Radiotherapy, Grenoble Alpes University Hospital, Grenoble, France; 6 Department of Urology and Kidney Transplantation, Grenoble Alpes University Hospital, Grenoble, France

**Keywords:** Bone SPECT/CT, 18F-FCH, PET/CT, Prostate cancer, Biochemical recurrence, Bone metastases, Molecular imaging, Oncology

## Abstract

**Background:**

To assess whether whole-body (WB) bone SPECT/CT provides additional diagnostic information over [18F]-FCH PET/CT for the detection of bone metastases in the setting of prostate cancer biochemical recurrence (PC-BR).

**Methods:**

Patients referred for a PC-BR and whom benefited from a WB bone SPECT/CT and FCH PET/CT were retrospectively included. Tests were classified as positive, equivocal, or negative for bone metastases. A best valuable comparator (BVC) strategy including imaging and follow-up data was used to determine the metastatic status in the absence of systematic histological evaluation.

**Results:**

Between January 2011 and November 2017, 115 consecutive patients with a PC-BR were evaluated. According to the BVC, 30 patients had bone metastases and 85 patients did not present with bone lesions. The sensitivity, specificity, positive and negative predictive values were respectively 86.7% [69.3–96.2], 98.8% [93.6–100.0], 96.3% [78.7–99.5], and 95.5% [89.4–98.1] for WB bone SPECT/CT and 93.3% [77.9–99.2], 100.0% [95.8–100.0], 100.0 and 97.7% [91.8–99.4] for FCH PET/CT. There was no significant difference in diagnostic accuracy of bone metastases between WB Bone SPECT/CT (AUC 0.824 [0.74–0.90]) and FCH PET/CT (AUC 0.829 [0.75–0.90], *p* = 0.41).

**Conclusion:**

Despite good performances for the diagnosis of bone metastases in PC-BR, WB bone SPECT/CT does not provide additive diagnostic information over concomitant FCH PET/CT.

## Introduction

Prostate cancer (PC) is the most common form of male cancer in Europe, representing about 400,000 new cases / year in Europe [1, 2] and the third leading cause of death in men over 50 years – old [3]. After initial post-diagnostic radical treatments, follow-up is performed through physical examination and blood prostate specific antigen (PSA) assessment [4].

However, biochemical recurrence (BR) occurs in 20–50% of patients within 10 years following local treatment [5]. BR can be defined by two consecutive rising PSA values > 0.2 ng/mL after radical prostatectomy (RP) or > 2 ng/mL above the nadir after external beam radiotherapy (EBRT) [6]. While PSA is a very sensitive tool to detect relapse, it is not suitable for the characterization of disease extension [7]. For patients presenting with biochemical recurrence, determining whether the disease is locally confined or systemically spread is of paramount importance for the selection of the most appropriate therapeutic strategy between local salvage treatment or systemic treatment.

Bone represents the second most common metastatic site of PC [8]. Bone metastasis represents an independent prognostic factor [9, 10] and an indication for a systemic therapy [11]. Bone scan (BS) scintigraphy and ^18^F-Choline (FCH) positron emission tomography (PET/CT) are currently available for routine diagnosis use in nuclear medicine for patients with biochemical recurrence of PC.

99 m-Technetium (^99m^Tc) labeled diphosphonate BS is a low-cost and widely available whole-body nuclear imaging test with good sensitivity [12]. However, planar BS has a relatively poor specificity due to the unspecific bone uptake related to osteoblastic activity and potentially leading to false positive results. BS is now supplemented with a single photon emission computed tomography fused with a low-dose CT scan (SPECT/CT) [13, 14], thatimproves the lesion-to-background ratio, allows anatomic lesion localization, removes the superimposition of anatomical structures and provides morphological data, thereby increasing the specificity and positive predictive value of BS [15, 16].

FCH currently represents the most commonly used PET tracer for the evaluation of patient with PC [17, 18]. Choline uptake is increased in PC cells due to tumoral cell proliferation and the subsequent increase in the activity of choline kinase [19, 20]. In addition to bone metastases, FCH PET/CT also allows the detection of local recurrence, lymph node or visceral metastases. FCH is the only available PET tracer considered as a reference in current clinical practice in France for the evaluation of patients with BR of PC [21]. Indeed, prostate-specific membrane antigen (PSMA) PET/CT, which might be considered now as the gold-standard in the setting of PC is still not available for routine use in France [22].

Bone scan is still currently performed by a number of institutions for the detection of bone metastases in BR of PC. Many studies discourage BS in patients with PSA < 10 ng/ml, because of insufficient diagnostic performance and very low detection rate [11, 23]. Nevertheless, these studies did not evaluate the potential of systematic whole-body (WB) bone SPECT/CT.

The aim of this study was to assess whether whole-body (WB) bone SPECT/CT provides additional diagnostic information over [18F]-FCH PET/CT for the detection of bone metastases in the setting of prostate cancer biochemical recurrence.

## Materials and methods

### Study design and patients

**Fig. 1:**
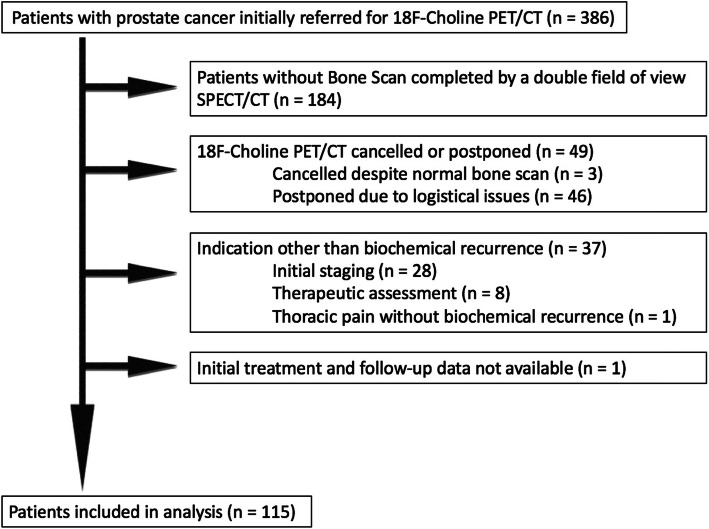
Flow chart of study population

We conducted a single-center retrospective study at the Grenoble Alpes University Hospital. From January 2011 to November 2017, a total of 386 consecutive patients were addressed in the nuclear medicine department to perform a FCH PET/CT in the setting of biochemical recurrence of PC. We included patients with biochemical recurrence of a histologically proven PC who benefited from whole-body BS coupled to a double field of view (FOV) SPECT/CT and a FCH PET/CT within 3 months, and for whom data on initial tumor and follow-up were available (Fig. 1). Such data were available since both tests were systematically performed for patients referred to our center for BR of PC. Data concerning the initial tumor (PSA at the diagnosis, Gleason score, TNM stage), the initial treatment of PC, the kinetics of PSA (PSA nadir, at least two values of PSA before the WB bone SPECT/CT with their date) and the follow up (clinical evaluation, biological and imaging data) were collected for all patients using our institution database. Data from patients followed in other institutions were collected through their corresponding prescribing physicians and medical biology laboratories by phone or email. The PSA doubling time was calculated using the most recent PSA values (with a minimum of 2 and up to 6 values) and the least squares fitting-exponential methodology (www.doubling-time.com). The study was approved by the local ethics committee.

### Whole-body bone SPECT/CT

Three hours after the intravenous administration of 7.4 MBq/Kg (0.2 mCi/Kg) (a minimum of 550 MBq) of 99mTc-methylene diphosphonate, planar whole-body images in anterior and posterior projections were obtained using a dual-head SPECT/CT camera Symbia-T2 (Siemens). A SPECT/CT of two FOV from the cervical spine to the proximal part of the thighs and therefore encompassing belts and the whole spine (whole-body bone SPECT/CT), was then performed in all patients. The camera was equipped with low-energy high-resolution (LEHR) collimators. Images were acquired on a 128 × 128 pixel matrix using 64 projections of 25 s each with 180° rotation for each camera head. SPECT data were reconstructed using an ordered subset expectation maximization (OSEM) algorithm (5 iterations over 8 subsets). A low-dose CT scan of the same zone was obtained for attenuation correction and anatomical localization. Data were acquired with exposure of 130 kV, a pitch of 1.85 with rotation time of 1.5 s and collimation of 2 × 2.5 mm, and reconstructed to images using 3 mm-thick slices.

### 18F-Choline PET/CT

PET/CT images were acquired on a PET∕CT Discovery-690 (GE Healthcare) after intravenous administration of 3.7 MBq/Kg (0.1 mCi/Kg) of FCH. Image acquisition consisting of a low dose CT scan followed by dynamic PET images of the pelvis over 10 min (10 × 1 min frames) was started immediately following tracer injection in order to overcome urinary bladder activity. Sixty minutes after injection, a whole-body PET/CT acquisition (from the skull to the proximal part of the thighs) was performed with an acquisition time of 2 min per bed positions. Unenhanced CT scan of the same zone was performed for attenuation correction and localization (120 kV, current modulation, 0.8 s/rotation, 3.75 mm reconstructed section thickness, 512 × 512 matrix). To limit the contribution of urinary elimination on FCH images, all patients received an intravenous injection of a diuretic (Furosemide 20 mg) and were asked to urinate before whole-body acquisition.

### Image interpretation

All the images from WB bone SPECT/CT and FCH PET/CT were analyzed by two blinded nuclear medicine physicians and were reviewed on a dedicated workstation. When a lesion was differentially classified, the expertise of a third nuclear medicine physician was used to resolve the discrepancy. Planar BS was visualized in anterior and posterior views whereas SPECT/CT and PET/CT acquisitions were evaluated in transverse, coronal and sagittal planes. The morphological data from CT scans were also studied for anatomical localization and detection of bone lesions. Each patient was classified as having malignant lesion, equivocal lesion, benign finding or a normal exam from WB bone SPECT/CT analysis. The lesions were classified as equivocal in the event that no consensus could be reached due to the lack of an alternative test. On FCH PET/CT, each focal or diffuse abnormal tracer uptake was classified as positive, equivocal or negative for bone involvement. Pathological focal uptake of FCH from the prostatic or visceral regions and lymph nodes was also studied.

As histological proof of bone metastases was absent in most cases, a best valuable comparator (BVC) was used as the gold standard, as previously described in other comparative imaging studies [24, 25]. BVC was determined collegially (nuclear medicine physician, urologist, oncologist, radiotherapist) using all available imaging data (current and follow-up) as well as the 6-month clinical and biological evolution.

### Statistical analysis

Continuous variables were reported as medians and interquartile ranges (IQRs) and categorical variables as frequencies and percentages. Baseline characteristics were compared according to WB bone SPECT/CT results using the chi-square test (or Fisher exact test when appropriate) for categorical variables and the nonparametric Kruskal-Wallis test for continuous variables. The comparison of WB bone SPECT/CT results was stratified by PSA doubling time classes, across the trigger PSA classes and the interaction was tested using the Mantel-Haenszel test.

The diagnostic performance parameters of WB bone SPECT/CT and FCH PET/CT compared to BVC (sensitivity [Se], specificity [Sp], positive predictive value [PPV], negative predictive value [NPV]) were presented using 95% confidence intervals (Cis) based on binomial distributions. Equivocal tests were considered negative upon single analysis since such tests did not prevent from performing additional investigations in order to identify secondary bone lesions. Receiver-operating characteristics (ROC) analysis were also obtained for each test with calculation of the area under the curve (AUC) and statistical comparison of ROC curves.

A two-sided *p* value < 0.05 was considered statistically significant. All analyses were performed using Stata Special Edition version 14.0 (Stata Corp, College Station, TX).

## Results

From January 2011 to November 2017, 115 patients were evaluated by both WB bone SPECT/CT and FCH PET/CT for a biochemical recurrence of PC. All data were available except the nadir PSA that was missing for two patients. Patients’ characteristics are summarized in Table 1*.* The median age of the population was 73.2 years (range 56–89). Ten (8.7%), 29 (25.2%), and 76 (66.1%) patients were respectively classified at low-risk, intermediate-risk and high-risk according to D’AMICO initial staging classification.

**Table 1:** Comparison of baseline patient’s characteristics based on whole-body bone SPECT/CT results

Characteristic	Total (*N* = 115)	Negative bone SPECT/CT (*N* = 88)	Positive bone SPECT/CT (*N* = 27)	*P* value
Age (years)				
Median ± (IQRs)	73.2 (56–89)	73.2 (67.2–80)	73.2 (66–81.3)	0.79
Initial PSA (ng/mL)				
Median ± (IQRs)	9.4 (7.1–18.4)	9.2 (7.2–17.3)	9.4 (6.5–33.5)	0.45
Initial PSA (ng/mL), class n (%)				0.18
0–10	65 (56.5)	49 (55.7)	16 (59.2)	
10.01–20	26 (22.6)	23 (26)	3 (11.1)	
> 20	24 (20.9)	16 (18)	8 (29.6)	
Tumor (T) stage, n (%)				0.10
T1 – T2a	34 (30.6)	30 (35.7)	4 (14.8)	
T2b	16 (14.4)	12 (14.3)	4 (14.8)	
T2c – T4	61 (55)	42 (50)	19 (70.4)	
Gleason score, n (%)				< 0.01
≤ 6	32 (27.8)	31 (35.2)	1 (3.7)	
7 (3–4)	29 (25.2)	24 (27.3)	5 (18.5)	
7 (4 + 3)	30 (26.1)	21 (23.9)	9 (33.3)	
≥ 8	24 (20.9)	12 (13.6)	12 (44.4)	
D’Amico classification, n (%)				0.09
Low-risk	10 (8.7)	10 (11.4)	0 (0)	
Intermediate-risk	29 (25.2)	24 (27.3)	5 (18.5)	
High-risk	76 (66.1)	54 (61.3)	22 (81.5)	
Initial treatment, n (%)				0.98
RP	13 (11.3)	10 (11.4)	3 (11.1)	
RP + EBRT	34 (29.6)	27 (30.7)	7 (25.9)	
With positive surgical margins	22 (19)	15 (17)	7 (26)	0.15
EBRT	50 (43.5)	34 (42)	13 (48.2)	
Other (Brachytherapy, HIFU)	18 (15.6)	14 (15.9)	4 (14.8)	
Adjuvant ADT	60 (52.2)	42 (47.7)	18 (66.7)	0.09
PSA nadir (ng/mL), median ± (IQRs)	0.1 (0.0–0.7)	0.1 (0.0–0.7)	0.1 (0.0–0.4)	0.62
Trigger PSA (ng/mL), median ± (IQRs)	5 (2.4–9.9)	4.4 (2.1–6.9)	12.5 (4.4–18.6)	< 0.001
Trigger PSA (ng/mL), class n (%)				< 0.001
PSA ≤10 ng /mL,	87 (75.7)	74 (84.1)	13 (48.2)	
PSA > 10 ng/mL	28 (24.4)	14 (15.9)	14 (51.9)	
PSAdt (months), median ± (IQRs)	6.1 (3.3–13.4)	7.6 (3.7–16.7)	3.8 (2.1–6)	< 0.001
PSAdt (months), n (%)				< 0.01
≤ 6 months	56 (48.7)	35 (39.8)	21 (77.8)	
> 6 months	59 (51.3)	53 (60.2)	6 (22.2)	

*Abbreviations*: *RP* Radical prostatectomy, *EBRT* External beam radiotherapy, *HIFU* High intensity focal ultrasound, *ADT* Androgen deprivation therapy, *IQRs* Interquartile ranges, *PSAdt* PSA doubling time

There were 13 patients whom were treated by RP only (11.3%), 50 patients were treated by EBRT only (43.5%), and 34 whom were treated by RP and EBRT (29.6%). Among the latter, 16 patients were treated with salvage EBRT and 18 patients were treated with adjuvant EBRT. Eighteen patients (15.6%) benefited from an alternative therapy (brachytherapy, high-intensity focused ultrasound HIFU). Twenty-two patients (35%) treated by RP had positive surgical margins (R1). At the time of the WB bone SPECT/CT the median PSA level (trigger PSA) was 5 ng/ml (2.4–9.9) with 87 patients (75.7%) having a PSA level ≤ 10 ng/ml and 28 (24.4%) having a PSA level >  10 ng/ml. The median PSAdt was 6.1 months (3.3–13.4) and 56 patients (48.7%) had a PSAdt ≤6 months.

According to the BVC 30 (26%) patients presented with bone metastases at this point while 85 (74%) did not. On the basis of WB bone SPECT/CT, 27 (23.5%) patients were classified as having bone metastases, 7 (6%) patients had equivocal bone lesions and 81 (70.5%) patients had a negative study or benign findings such as degenerative lesions. Compared with BVC, 26 true positives, 1 false positive, 4 false negatives and 84 true negatives tests were obtained. Sensitivity, specificity, positive and negative predictive values were respectively 86.7% [69.3–96.2], 98.8% [93.6–100.0], 96.3% [78.7–99.5], and 95.5% [89.4–98.1] for WB bone SPECT/CT.

With FCH PET/CT, 28 (24%) patients were classified as having malignant lesions on the skeleton. Eighty-five patients (76%) had no focal or diffuse uptake on the skeleton and were therefore considered has having a negative test. There were two equivocal findings. Compared with BVC, 28 true positives, no false positive, 2 false negatives and 85 true negatives were observed. Sensitivity, specificity, positive and negative predictive values were respectively 93.3% [77.9–99.2], 100.0% [95.8–100.0], 100.0 and 97.7% [91.8–99.4] for FCH PET/CT (Tables 2 and 3).

**Table 2:** Results of whole-body bone SPECT/CT and FCH PET/CT for the different sites evaluated

Relapse site N (%)	Bone			Lymph node	Local	Total
	Positive	Negative	Equivocal	Positive	Positive	Positive
Bone SPECT/CT	27 (23.5%)	81 (70.5%)	7 (6%)	/	/	27 (23.5%)
FCH PET/CT	28 (24%)	85 (74%)	2 (1.5%)	45 (41.5%)	35 (32%)	85 (74%)
BVC	30 (26%)	85 (74%)	0 (0%)

**Table 3:** Diagnostic performances of whole-body bone SPECT/CT and FCH PET/CT in comparison with the best valuable comparator

	Bone SPECT/CT (*N* = 115)	FCH PET/CT (N = 115)
True positive	*N* = 26	*N* = 28
True negative	*N* = 84	*N* = 85
False positive	*N* = 1	*N* = 0
False negative	*N* = 4	*N* = 2
Sensitivity	86.7% (69.3–96.2)	93.3 (77.9–99.2) %
Specificity	98.8% (93.6–100.0)	100.0 (95.8–100.0) %
Positive predictive value	96.3% (78.7–99.5)	100.0%
Negative predictive value	95.5% (89.4–98.1)	97.7 (91.8–99.4) %
AUC	0.824 (0.74–0.90)	0.829 (0.75–0.90)

95% CIs are given in parentheses

*AUC* Area under the curve

FCH PET/CT also identified hypermetabolic lymph nodes in 45 (41.5%) patients and local recurrence either in the prostate gland or bed in 35 (32%) patients. One patient had a metastatic lung extension in addition to bone involvement. FCH PET/CT identified the site of relapse for 85 patients among the 115 patients, which represents a detection rate of 74%.

Overall, there was no significant difference in diagnostic accuracy of bone metastases between WB Bone SPECT/CT (AUC 0.824 [0.74–0.90]) and FCH PET/CT (AUC 0.829 [0.75–0.90], *p* = 0.41).

Among patients with bone lesions on WB bone SPECT/CT (true positive), 9 had a single bone lesion, 8 had an oligometastatic disease (between 2 and 5 lesions) and 9 had a high bone metastatic volume (> 5 lesions). More bone lesions were detected using FCH PET/CT but only five patients switched group. Specifically, 2 false-negative WB bone SPECT/CT had bone involvement (single lesion and high metastatic tumoral volume) identified using FCH PET/CT; 1 patient was reclassified from single to oligometastatic lesions; 1 patient with equivocal lesions upon WB Bone SPECT/CT presented with oligometastatic lesions upon FCH PET/CT; and 1 patient was a WB bone SPECT/CT false-positive (Table 1).

### Characteristics associated with the positivity of whole-body bone SPECT/CT

In univariate analysis, patients with a positive WB bone SPECT/CT had a significantly higher initial Gleason score (*p* < 0.01). The median PSA value at the time of WB bone SPECT/CT was significantly higher for patients with positive WB bone SPECT/CT (12.5 ng/mL) compared with those with negative WB bone SPECT/CT (4.4 ng/mL, *p* < 0.001). A PSA threshold of 10 ng/ml was significantly associated with positive WB bone SPECT/CT (*p* < 0.001). The median PSAdt of patients with positive WB bone SPECT/CT was significantly shorter than those with negative test (3.8 versus 7.6 months respectively, *p* < 0.001). The PSAdt threshold of 6 months was predictive of a positive WB bone SPECT/CT (*p* < 0.01) (Table 4). The identification of patients with high bone metastatic volume was challenging when these pre-test predictive factors were used. Indeed, 4 out of 9 patients with high bone metastatic volume had a Gleason score ≥ 8, 6 had a pre-test PSA >  10 ng/mL, and 7 had a PSAdt ≤6 months while only 1 patient was positive for the 3 identified predictive factors.

**Table 4:** Comparison of whole-body bone SPECT/CT results between PSA doubling time classes according to trigger PSA classes (*p* = 0.01 for global interaction)

PSA ≤10 ng/ml, *N* = 87				
Bone SPECT/CT result	Negative (*N* = 74)	Positive (*N* = 13)	OR (CI95)	*p*
PSAdt > 6 months	49 (92.5)	4 (7.5)	reference	0.03
PSAdt ≤6 months	25 (73.5)	9 (26.5)	4.4 (1.1–21.2)	
PSA > 10 ng/ml, *N* = 28				
Bone SPECT/CT result	Negative (*N* = 14)	Positive (*N* = 14)	OR (CI95)	*p*
PSAdt > 6 months	4 (66.7)	2 (33.3)	reference	0.32
PSAdt ≤6 months	10 (45.5)	12 (54.5)	2.4 (0.3–30.9)	

### Detection rate and influence of PSA and PSA kinetics

Overall, 87 patients had a PSA level lower than 10 ng/mL before the WB bone SPECT/CT, of whom 13 (15%) had a positive WB bone SPECT/CT. In those patients with a PSA < 10 ng/mL (median PSA = 2.96 ng/ml [2.62–5.45]), 9/13 (69%) patients with a positive WB bone SPECT/CT had a PSAdt ≤6 months (median = 2.63 months [2.17–4.73]). On the other hand, 28 patients had a PSA level higher than 10 ng/mL before WB bone SPECT/CT, including 14/28 (50%) patients with positive WB bone SPECT/CT. In this group, 12 patients (86%) with a positive WB bone SPECT/CT had a PSAdt ≤6 months.

Patients with a positive WB bone SPECT/CT had higher rates of PSAdt ≤6 months (77.8% vs. 39.8%). Interestingly, a significant interaction was observed between trigger PSA and PSAdt for the odds of the WB bone SPECT/CT results: compared with patients with a PSAdt > 6 months, patients with a shorter PSAdt and with a trigger PSA lower than 10 ng/ml had higher rates of positive WB bone SPECT/CT (OR = 4.4, CI95 [1.1–21.2], *p* = 0.03) (Table 4).

**Table 5:** Whole-body bone SPECT/CT detection rates according to pre-test PSA classes

PSA class	Bone metastatic patient N (%)
> 10 ng/mL	14/28 (50%)
2–10 ng/mL	11/65 (17%)
1–2 ng/mL	1/12 (8%)
< 1 ng/mL	1/10 (10%)
Total	27/115 (23%)

The numbers and percentages are expressed in relation to the total number of patients in each PSA class

**Fig. 2:**
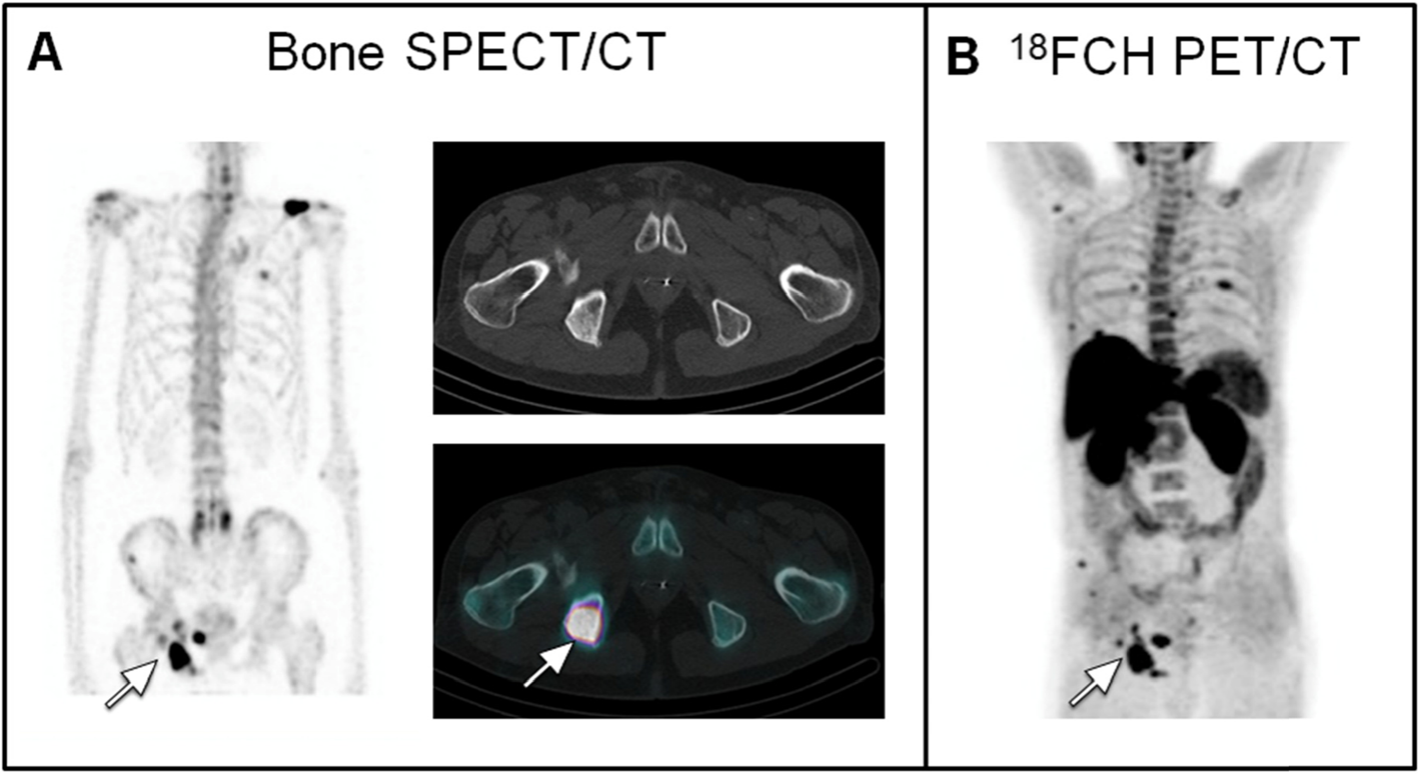
A 75 years-old male patient with a high-risk prostate cancer (initial stage T3N0, Gleason score 7 (3 + 4), a baseline PSA level of 8 ng/mL), initially treated by EBRT and adjuvant hormonotherapy. The patient presented with a biochemical recurrence after 4 years of follow-up (PSA: 2.77 ng/mL; nadir: 0.03 ng/mL; PSA doubling time 2.17 months). A whole-body bone SPECT/CT was performed (**a** Maximum Intensity Projection (MIP) image and axial plane) and multiple osteoblastic lesions interesting the pelvis (white arrow), the ribs, the right humeral head were observed. The patient had a recent fracture of the left clavicle. FCH PET/CT (see the image from (**b**) with MIP image) confirmed the presence of multiple bone metastases (of which some lesions not visualized in bone SPECT/CT), with no lesion on the prostate bed or the lymph node. A treatment by hormonotherapy was introduced

**Fig. 3:**
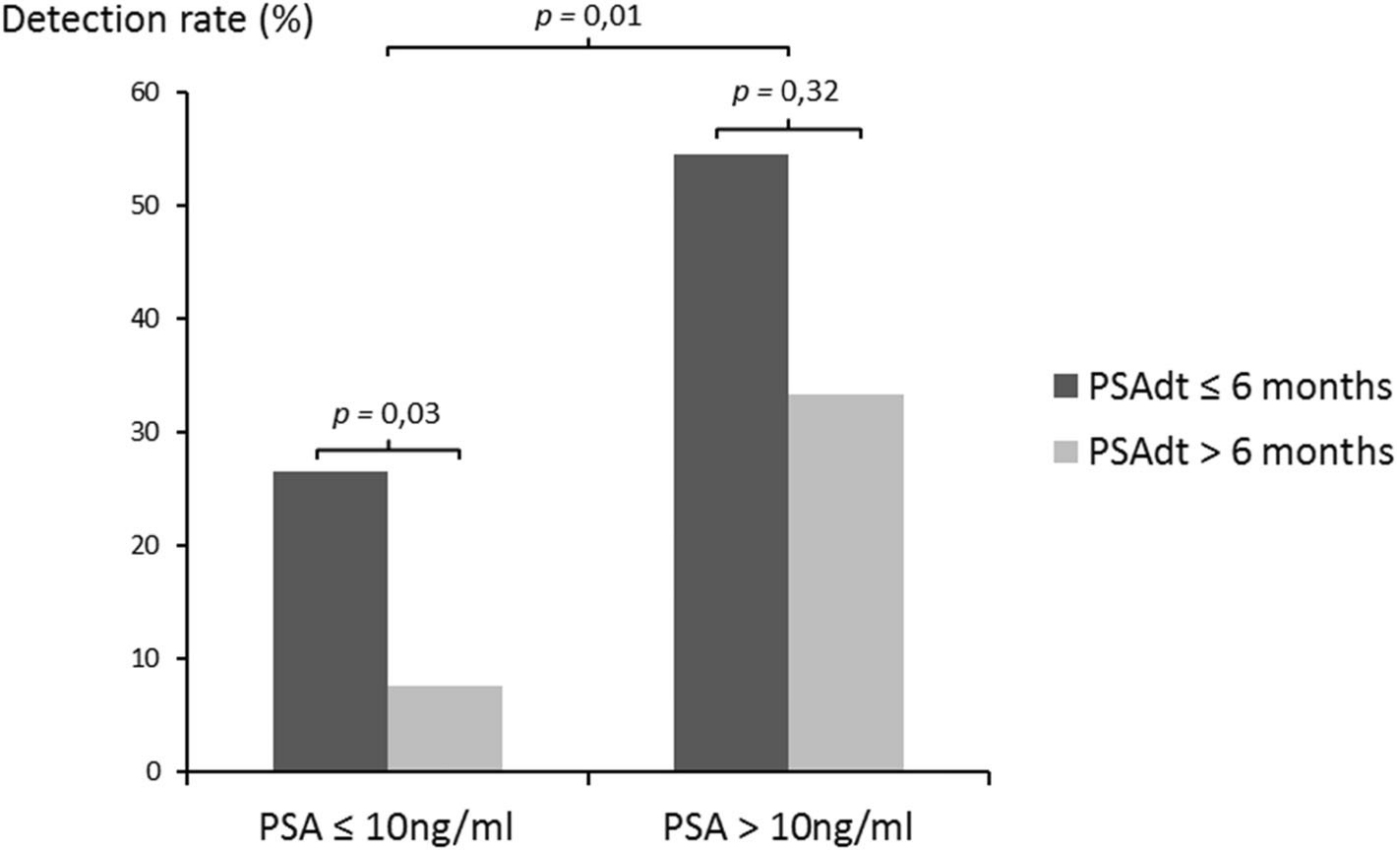
Detection rate of bone lesion on whole-body bone SPECT/CT according to PSA trigger and PSA doubling time

Indeed, the detection rate in this patient subgroup was 26.5% (9/34 patients) as compared with 7.5% (4/53 patients) in the subgroup of patients with PSA < 10 ng/ml and PSAdt > 6 months (Figs. 2 and 3). The detection rate of bone metastases was 50% when the PSA > 10 ng/mL and decreased for PSA classes below 10 ng/mL, valued at 17% for PSA 2–10 ng/ml, 8% for PSA between 1 and 2 ng/ml (one positive patient), 10% for PSA < 1 ng/ml (one positive patient) (Tables 5 and 6).

**Table 6:** Distribution of the number of bone lesions identified by Bone SPECT/CT and FCH PET/CT

Lesions	Bone SPECT/CT	FCH PET/CT
0	89 (77%)	87 (75%)
1	9 (8%)	8 (7%)
2–5	8 (7%)	10 (9%)
> 5	9 (8%)	10 (9%)

Percentages are expressed in relation to the total number of patients. Equivocal bone lesions were classified as negative

## Discussion

The main result of this study is that bone scan with systematic double FOV SPECT/CT (WB bone SPECT/CT) has good diagnostic performances for the detection of bone metastases in comparison to FCH PET/CT in patients with BR of PC, but that it does not provide additional diagnostic information. To the best of our knowledge, this is the first study to evaluate the diagnostic performances of bone scan complemented by a systematic WB bone SPECT/CT in patients with BR of PC.

### Clinical setting

The detection of bone metastases induces radical changes in the prognosis and therapeutic management of patients with BR of PC, thereby justifying the clinical relevance of powerful corresponding diagnostic tools. WB bone SPECT/CT is a low-cost, widely available and frequently used first intention clinical test for the detection of bone metastases in PC. However, the use of WB bone SPECT/CT has been recently questioned, especially in patients with a PSA < 10 ng/mL [4]. FCH PET/CT is currently considered as the reference tracer for the evaluation of patients with BR of PC in France [21]. In the present study, we have retrospectively evaluated the diagnostic accuracy of WB bone SPECT/CT performed prior to FCH PET/CT in 115 consecutive patients with BR of PC after radical initial treatment. The median PSA value (5 ng/ml [2.4–9.9]) observed in the present study was lower than values from previously published studies addressing the performances of bone scan in biochemically recurrent disease [26–28]. Although such value might appear elevated with respect to the diagnostic criteria for BR, it is likely explained by the fact that PSA was determined at the time considered as the most relevant for recurrence localization using FCH PET/CT, as indicated by a FCH PET/CT detection rate (74%) similar to that observed in previously published studies [21].

### Diagnostic performance of whole-body bone SPECT/CT

Among the papers published studying the performance of bone scan in prostate cancer, none evaluated the performances of systematic whole-body bone SPECT/CT in BR of PC. These previously published studies rather evaluated the performances of planar bone scintigraphy – an out-of-date procedure [29, 30], or the performances of non-systematic SPECT/CT indicated on the basis of planar scintigraphic findings [31, 32].

Our study confirms the good diagnostic performances of WB bone SPECT/CT in prostate BR with sensitivity, specificity, positive and negative predictive values of 86.7% [69.3–96.2], 98.8% [93.6–100.0], 96.3% [78.7–99.5], and 95.5% [89.4–98.1] when equivocal WB bone SPECT/CT were considered as negative, in comparison to the BVC and regardless of the PSA level.

Picchio et al. classified equivocal results from scintigraphy (27%) as either positive or negative, inducing a quite large range for sensitivity [70–100%] and specificity [75–100%] [30]. However, an optimal strategy taking into account both the medical and medico-economical perspectives obviously requires the classification of equivocal studies as negative since all equivocal cases require an additional test to establish a reliable diagnosis.

Accordingly, when considering equivocal WB bone SPECT/CT as negative, the results of Picchio et al. point at the suboptimal performances of planar bone scintigraphy (sensitivity, 70%; specificity, 100%) with respect to those observed in the present study using WB bone SPECT/CT (sensitivity, 86.7%; specificity, 98.8%). Overall, the diagnostic performances of WB bone SPECT/CT are superior to those described in the literature for planar BS [33–38]. The rate of positive WB bone SPECT/CT that we observed (27 patients, 23.5%) appears relatively high compared to that found in previous studies with planar BS (6 to 14%) [26, 28, 29] and similar to those of Moreira et al. (20–26%) [39]. However, details regarding the acquisition protocol (planar with or without SPECT/CT) were not provided by the authors of the latter study. Furthermore, we noticed a relatively low equivocal examination rate, with only 7 patients (6%) classified as doubtful for the presence of bone metastases, while the remaining patients were immediately categorized as positive or negative. These results are in accordance with those of Helyar et al. who reported an 8% rate of equivocal lesions according to SPECT/CT and a significantly reduced rate of equivocal lesions in comparison with that of planar BS alone [40].

No significant difference was observed for the detection of bone metastases in comparison with FCH PET/CT (AUC 0.824 for WB Bone SPECT/CT vs 0.829 for FCH PET/CT, *p* = 0.41). These AUCs are comparable with those obtained by Pyka et al. [24] for bone SPECT/CT, even though a small number of patients benefited from a bone SPECT/CT in this study. More bone lesions were detected by FCH PET/CT than by WB bone SPECT/CT in the present study. However, the data from both tests were mostly in accordance regarding the classification of bone metastasis patients into the subgroups of single metastatic lesion, oligometastatic lesions, and patients with high bone metastatic volume.

Our results are deprived of any ambiguity since 74% of patients (85/115) benefited from FCH PET/CT which allowed the detection of one relapse site while only 22.6% of patients (26/115) benefited from whole-body bone SPECT/CT for the detection of bone metastases, with an even greater number of bone metastasis patients identified by FCH PET/CT (28/115). Even though WB bone SPECT/CT displays good performances for patient classification, it does not allow the assessment of lymph node or visceral metastatic disease. Such a knowledge is however essential in order to determine the appropriate treatment.

### Perspective

A current clinical problem consists in the detection of early recurrence which may still be accessible to local therapy and which may develop in the setting of low PSA levels. In the present study, the detection rate of bone lesions by WB bone SPECT/CT was low for PSA levels between 1 and 2 ng/mL and < 1 ng/mL, suggesting the suboptimal performances of the test in this setting.

PSMA has recently become a very promising target for the diagnostic and therapeutic management of patients with PC. PSMA-PET/CT was shown to outperform planar BS and bone SPECT/CT for the detection of bone metastases [24, 40] and FCH PET/CT for the number of detected bone metastases and for the overall number of identified recurrence sites [22], especially in patients presenting with low, < 2 ng/mL PSA levels. Indeed, the AUC of WB bone SPECT/CT and FCH PET/CT were comparable in our study and both were equivalent to the AUC observed by Pyka et al. [24] for WB bone SPECT/CT whereas the authors found a significantly better AUC for PSMA PET/CT.

### Limitations

There are several limitations to the present study. First, this is a single-centered and retrospective study.

However, the bias caused by retrospective design remains acceptable in comparison with previously published data on planar bone scan scintigraphy versus choline PET/CT (^11^C or ^18^F). For instance, patients being included over an initial population in our study represented (115/386) 29.8% versus (78/6266) 1.2% in the study by Picchio et al. [30].

Second, patients were referred for FCH PET/CT in the setting of BR of PC, implying the presence of high PSA levels with respect to the biological definition of BR.

A third limitation to be considered is the choice of FCH as the comparison test rather than the gold-standard PSMA-PET/CT. However, PSMA-PET/CT is not available yet in France and could therefore not be used as a reference in our study. The implications of our study are therefore limited to those countries with no access yet to PSMA-PET/CT in clinical routine or to those centers with no access to a 68Ga generator. Such situations are unfortunately still dominant worldwide, which in our opinion underlines the relevance of our study.

Fourth, systematic histological confirmation of bone lesions is impracticable and unethical when not impacting patient management. In the absence of histological evidence, others have used morphological or functional imaging modalities and follow-up as gold standards [32, 35, 41, 42]. In the present study, the BVC includes some clinical judgment or interpretation and is not totally independent of our diagnostic tests, which may lead to an inclusion bias and an overestimation of diagnostic performances. However, the BVC as a gold-standard appears as the best alternative to systematic histological analysis.

Finally, the choice of the frequency and timing of PSA measurements were left to treating physicians, which could have led to more variability in the assessment of PSAdt while however reflecting more accurately the clinical reality.

## Conclusion

Despite good performances for the diagnosis of bone metastases in prostate cancer biochemical recurrence, whole-body bone SPECT/CT does not provide additive diagnostic information over concomitant FCH PET/CT.

## Data Availability

Applicable if necessary.

## Abbreviations

PC: Prostate cancer
RP: Radical prostatectomy
EBRT: External beam radiotherapy
PSA: Prostate specific antigen
BR: Biochemical recurrence
BS: Bone scan
FCH: 18F-Choline
PET: Positron emission tomography
SPECT: Single photon emission computed tomography
PSMA: Prostate specific membrane antigen
WB: Whole-body
FOV: Field of view
BVC: Best valuable comparator
IQRs: Interquartile ranges
Cis: Confidence intervals
ROC: Receiver-operating characteristics
AUC: Area under the curve

## References

[1] Ferlay J, Steliarova-Foucher E, Lortet-Tieulent J, Rosso S, Coebergh JWW, Comber H (2013). Cancer incidence and mortality patterns in Europe: estimates for 40 countries in 2012. Eur J Cancer Oxf Engl 1990.

[2] Rébillard X, Grosclaude P, Leone N, Velten M, Coureau G, Villers A (2013). Incidence and mortality of urological cancers in 2012 in France. Progres En Urol J Assoc Francaise Urol Soc Francaise Urol.

[3] Wong MCS, Goggins WB, Wang HHX, Fung FDH, Leung C, Wong SYS (2016). Global incidence and mortality for prostate cancer: analysis of temporal patterns and trends in 36 countries. Eur Urol.

[4] Mottet N, Bellmunt J, Bolla M, Briers E, Cumberbatch MG, De Santis M (2017). EAU-ESTRO-SIOG guidelines on prostate cancer. Part 1: screening, diagnosis, and local treatment with curative intent. Eur Urol.

[5] Han M, Partin AW, Zahurak M, Piantadosi S, Epstein JI, Walsh PC (2003). Biochemical (prostate specific antigen) recurrence probability following radical prostatectomy for clinically localized prostate cancer. J Urol.

[6] Roach M, Hanks G, Thames H, Schellhammer P, Shipley WU, Sokol GH (2006). Defining biochemical failure following radiotherapy with or without hormonal therapy in men with clinically localized prostate cancer: recommendations of the RTOG-ASTRO Phoenix Consensus Conference. Int J Radiat Oncol Biol Phys.

[7] Johnson AC, Dugué AE, Silva M, Moise L, Tillou X, Joly F (2016). Predictive factors of 18F-choline PET/CT positivity in patients with prostate cancer recurrence after radiation therapy: is the impact of PSA nadir underestimated?. EJNMMI Res.

[8] Bubendorf L, Schöpfer A, Wagner U, Sauter G, Moch H, Willi N (2000). Metastatic patterns of prostate cancer: an autopsy study of 1,589 patients. Hum Pathol.

[9] Shou J, Zhang Q, Wang S, Zhang D (2018). The prognosis of different distant metastases pattern in prostate cancer: a population based retrospective study. Prostate.

[10] de Voogt HJ, Suciu S, Sylvester R, Pavone-Macaluso M, Smith PH, de Pauw M (1989). Multivariate analysis of prognostic factors in patients with advanced prostatic cancer: results from 2 European Organization for Research on Treatment of Cancer trials. J Urol.

[11] Cornford P, Bellmunt J, Bolla M, Briers E, De Santis M, Gross T (2017). EAU-ESTRO-SIOG guidelines on prostate cancer. Part II: treatment of relapsing, metastatic, and castration-resistant prostate cancer. Eur Urol.

[12] Brenner AI, Koshy J, Morey J, Lin C, DiPoce J (2012). The bone scan. Semin Nucl Med.

[13] Buck AK, Nekolla S, Ziegler S, Beer A, Krause BJ, Herrmann K (2008). SPECT/CT. J Nucl Med Off Publ Soc Nucl Med.

[14] Keidar Z, Israel O, Krausz Y (2003). SPECT/CT in tumor imaging: technical aspects and clinical applications. Semin Nucl Med.

[15] Savelli G, Maffioli L, Maccauro M, De Deckere E, Bombardieri E (2001). Bone scintigraphy and the added value of SPECT (single photon emission tomography) in detecting skeletal lesions. Q J Nucl Med Off Publ Ital Assoc Nucl Med AIMN Int Assoc Radiopharmacol IAR.

[16] Giovanella L, Castellani M, Suriano S, Ruberto T, Ceriani L, Tagliabue L (2011). Multi-field-of-view SPECT is superior to whole-body scanning for assessing metastatic bone disease in patients with prostate cancer. Tumori.

[17] Umbehr MH, Müntener M, Hany T, Sulser T, Bachmann LM (2013). The role of 11C-choline and 18F-fluorocholine positron emission tomography (PET) and PET/CT in prostate cancer: a systematic review and meta-analysis. Eur Urol.

[18] Bauman G, Belhocine T, Kovacs M, Ward A, Beheshti M, Rachinsky I (2012). 18F-fluorocholine for prostate cancer imaging: a systematic review of the literature. Prostate Cancer Prostatic Dis.

[19] Ackerstaff E, Glunde K, Bhujwalla ZM (2003). Choline phospholipid metabolism: a target in cancer cells?. J Cell Biochem.

[20] de Jong IJ, Pruim J, Elsinga PH, Vaalburg W, Mensink HJA (2002). Visualization of prostate cancer with 11C-choline positron emission tomography. Eur Urol.

[21] Evangelista L, Briganti A, Fanti S, Joniau S, Reske S, Schiavina R (2016). New clinical indications for (18)F/(11)C-choline, new tracers for positron emission tomography and a promising hybrid device for prostate cancer staging: a systematic review of the literature. Eur Urol.

[22] Morigi JJ, Stricker PD, van Leeuwen PJ, Tang R, Ho B, Nguyen Q (2015). Prospective comparison of 18F-Fluoromethylcholine versus 68Ga-PSMA PET/CT in prostate cancer patients who have rising PSA after curative treatment and are being considered for targeted therapy. J Nucl Med Off Publ Soc Nucl Med.

[23] Beresford MJ, Gillatt D, Benson RJ, Ajithkumar T (2010). A systematic review of the role of imaging before salvage radiotherapy for post-prostatectomy biochemical recurrence. Clin Oncol R Coll Radiol G B.

[24] Pyka T, Okamoto S, Dahlbender M, Tauber R, Retz M, Heck M (2016). Comparison of bone scintigraphy and 68Ga-PSMA PET for skeletal staging in prostate cancer. Eur J Nucl Med Mol Imaging.

[25] Zacho HD, Nielsen JB, Afshar-Oromieh A, Haberkorn U, deSouza N, De Paepe K (2018). Prospective comparison of 68Ga-PSMA PET/CT, 18F-sodium fluoride PET/CT and diffusion weighted-MRI at for the detection of bone metastases in biochemically recurrent prostate cancer. Eur J Nucl Med Mol Imaging.

[26] Dotan ZA, Bianco FJ, Rabbani F, Eastham JA, Fearn P, Scher HI (2005). Pattern of prostate-specific antigen (PSA) failure dictates the probability of a positive bone scan in patients with an increasing PSA after radical prostatectomy. J Clin Oncol Off J Am Soc Clin Oncol.

[27] Moreira DM, Howard LE, Sourbeer KN, Amarasekara HS, Chow LC, Cockrell DC (2015). Predicting bone scan positivity in non-metastatic castration-resistant prostate cancer. Prostate Cancer Prostatic Dis.

[28] Okotie OT, Aronson WJ, Wieder JA, Liao Y, Dorey F, DeKERNION JB (2004). Predictors of metastatic disease in men with biochemical failure following radical prostatectomy. J Urol.

[29] Kane CJ, Amling CL, Johnstone PAS, Pak N, Lance RS, Thrasher JB (2003). Limited value of bone scintigraphy and computed tomography in assessing biochemical failure after radical prostatectomy. Urology.

[30] Picchio M, Spinapolice EG, Fallanca F, Crivellaro C, Giovacchini G, Gianolli L (2012). [11C]choline PET/CT detection of bone metastases in patients with PSA progression after primary treatment for prostate cancer: comparison with bone scintigraphy. Eur J Nucl Med Mol Imaging.

[31] Shen G, Deng H, Hu S, Jia Z (2014). Comparison of choline-PET/CT, MRI, SPECT, and bone scintigraphy in the diagnosis of bone metastases in patients with prostate cancer: a meta-analysis. Skelet Radiol.

[32] Fuccio C, Castellucci P, Schiavina R, Guidalotti PL, Gavaruzzi G, Montini GC (2012). Role of 11C-choline PET/CT in the re-staging of prostate cancer patients with biochemical relapse and negative results at bone scintigraphy. Eur J Radiol.

[33] Palmedo H, Marx C, Ebert A, Kreft B, Ko Y, Türler A (2014). Whole-body SPECT/CT for bone scintigraphy: diagnostic value and effect on patient management in oncological patients. Eur J Nucl Med Mol Imaging.

[34] Schirrmeister H, Guhlmann A, Elsner K, Kotzerke J, Glatting G, Rentschler M (1999). Sensitivity in detecting osseous lesions depends on anatomic localization: planar bone scintigraphy versus 18F PET. J Nucl Med Off Publ Soc Nucl Med.

[35] Garcia JR, Moreno C, Valls E, Cozar P, Bassa P, Soler M (2015). Diagnostic performance of bone scintigraphy and (11)C-choline PET/CT in the detection of bone metastases in patients with biochemical recurrence of prostate cancer. Rev Espanola Med Nucl E Imagen Mol.

[36] Takesh M, Odat Allh K, Adams S, Zechmann C (2012). Diagnostic role of (18)F-FECH-PET/CT compared with bone scan in evaluating the prostate cancer patients referring with biochemical recurrence. ISRN Oncol.

[37] Even-Sapir E, Metser U, Mishani E, Lievshitz G, Lerman H, Leibovitch I (2006). The detection of bone metastases in patients with high-risk prostate cancer: 99mTc-MDP Planar bone scintigraphy, single- and multi-field-of-view SPECT, 18F-fluoride PET, and 18F-fluoride PET/CT. J Nucl Med Off Publ Soc Nucl Med.

[38] Moreira DM, Cooperberg MR, Howard LE, Aronson WJ, Kane CJ, Terris MK, et al. Predicting bone scan positivity after biochemical recurrence following radical prostatectomy in both hormone-naive men and patients receiving androgen-deprivation therapy: results from the SEARCH database. Prostate Cancer Prostatic Dis. 2014;17:91–6. 10.1038/pcan.2013.59 PMC4564861 24418913

[39] Helyar V, Mohan HK, Barwick T, Livieratos L, Gnanasegaran G, Clarke SEM (2010). The added value of multislice SPECT/CT in patients with equivocal bony metastasis from carcinoma of the prostate. Eur J Nucl Med Mol Imaging.

[40] Simsek DH, Sanli Y, Civan C, Engin MN, Isik EG, Ozkan ZG (2020). Does bone scintigraphy still have a role in the era of 68 Ga-PSMA PET/CT in prostate cancer?. Ann Nucl Med.

[41] Picchio M, Briganti A, Fanti S, Heidenreich A, Krause BJ, Messa C (2011). The role of choline positron emission tomography/computed tomography in the management of patients with prostate-specific antigen progression after radical treatment of prostate cancer. Eur Urol.

[42] Strobel K, Burger C, Seifert B, Husarik DB, Soyka JD, Hany TF (2007). Characterization of focal bone lesions in the axial skeleton: performance of planar bone scintigraphy compared with SPECT and SPECT fused with CT. AJR Am J Roentgenol.

